# Transitional care interventions reduce unplanned hospital readmissions in high-risk older adults

**DOI:** 10.1186/s12913-018-3771-9

**Published:** 2018-12-12

**Authors:** Kathleen Finlayson, Anne M. Chang, Mary D. Courtney, Helen E. Edwards, Anthony W. Parker, Kyra Hamilton, Thu Dinh Xuan Pham, Jane O’Brien

**Affiliations:** 1 School of Nursing, Institute of Health and Biomedical Innovation, Queensland University of Technology, Brisbane, Australia; 2 Brisbane, Australia; 3 Faculty of Health, Institute of Health and Biomedical Innovation, Queensland University of Technology, Brisbane, Australia; 4 School of Exercise and Nutrition Sciences, Institute of Health and Biomedical Innovation, Queensland University of Technology, Brisbane, Australia; 5 School of Applied Psychology, Menzies Health Institute Queensland, Griffith University, Brisbane, Australia; 6 School of Cultural and Professional Learning, Faculty of Education, Queensland University of Technology, Brisbane, Australia; 7 School of Health Sciences, University of Tasmania, Launceston, Australia

**Keywords:** Hospital readmission, Older adults, Randomised controlled trial, Transitional care

## Abstract

**Background:**

Acute hospital services account for the largest proportion of health care system budgets, and older adults are the most frequent users. As a result, older people who have been recently discharged from hospital may be at greater risk of readmission. This study aims to evaluate the comparative effectiveness of transitional care interventions on unplanned hospital readmissions within 28 days, 12 weeks and 24 weeks following hospital discharge.

**Method:**

The present study was a randomised controlled trial (ACTRN12608000202369). The trial involved 222 participants who were recruited from medical wards in two metropolitan hospitals in Australia. Participants were eligible for inclusion if they were aged 65 years and over, admitted with a medical diagnosis and had at least one risk factor for readmission. Participants were randomised to one of four groups: standard care, exercise program only, Nurse Home visit and Telephone follow-up (N-HaT), or Exercise program and Nurse Home visit and Telephone follow-up (ExN-HaT). Socio-demographics, health and functional ability were assessed at baseline, 28 days, 12 weeks and 24 weeks. The primary outcome measure was unplanned hospital readmission which was defined as any hospital admission for an unforeseen or unplanned cause.

**Results:**

Participants in the ExN-HaT or the N-HaT groups were 3.6 times and 2.6 times respectively significantly less likely to have an unplanned readmission 28 days following discharge (ExN-HaT group HR 0.28, 95% CI 0.09–0.87, *p* = 0.029; N-HaT group HR 0.38, 95% CI 0.13–1.07, *p* = 0.067). Participants in the ExN-HaT or the N-HaT groups were 2.13 and 2.63 times respectively less likely to have an unplanned readmission in the 12 weeks after discharge (ExN-HaT group HR 0.47, 95% CI 0.23–0.97, *p* = 0.014; N-HaT group HR 0.38, 95% CI 0.18–0.82, *p* = 0.040). At 24 weeks after discharge, there were no significant differences between groups.

**Conclusion:**

Multifaceted transitional care interventions across hospital and community settings are beneficial, with lower hospital readmission rates observed in those receiving more transitional intervention components, although only in first 12 weeks.

**Trial registration:**

Australian and New Zealand Clinical Trial Registry (ACTRN12608000202369).

## Background

The ageing profile of populations worldwide presents a significant challenge to the delivery of health services. Acute hospital services account for the largest proportion of health care system budgets, and older adults are the most frequent users, both for initial hospital admissions and for readmissions [1]. Determining optimal transitional care for older adults following hospitalisation has not yet been achieved. Care for older adults with co-morbidities is often poorly coordinated. This is reflected in a steady increase in the rates of ‘preventable’ hospitalisations (e.g. chronic conditions, complications), a serious and costly issue [1].

In the USA, comprehensive discharge planning and follow-up interventions have demonstrated short-term reductions in readmissions of at-risk older people [2, 3]. However, systematic reviews on discharge planning [4], follow-up care [5] and exercise interventions [6, 7] have shown conflicting results with only small effects on readmissions and limited evidence on improved health outcomes [4]. Another limitation is inconsistency between definition of readmissions, i.e., all readmissions, or only unplanned readmissions, and/or differing time frames [8].

Systematic reviews [9, 10] on interventions to reduce hospital readmissions of older people indicate that no single intervention is effective in reducing older people’s readmission and that a more holistic approach is required. A review of comprehensive geriatric assessment, i.e. multidimensional assessment of health and capabilities in order to develop an integrated plan, found no clear evidence of benefits in mortality, readmissions, institutionalization, functional ability, quality of life and/or cognition for those who were discharged within 72 h from hospital settings [11]. Batty’s review [9] concluded that the most effective models in preventing older people being admitted to hospital are provided by established, integrated teams in the patient’s home.

In Australia, patients are predominantly discharged from hospital into the care of their primary care General Practitioner. Additional home support services for those with multiple comorbid conditions and/or decreased functional ability is provided by a range of poorly integrated community services, both privately and publicly funded. An earlier study conducted by this team demonstrated significant reductions in unplanned health service use following discharge, showing a 43% reduction in unplanned readmissions and ~ 20% improvement in functional ability [12, 13] following the implementation of a 6 month multifaceted transitional care intervention across hospital and community settings for older people at risk of poor outcomes following hospitalisation. However, the comparative effectiveness of each of the interventions (i.e. hospital and home exercise strategies, and/or nurse in-home visits and telephone follow-up) on outcomes of hospital readmission, unplanned health service use, functional ability and quality of life has not been evaluated in our previous study or in the literature.

The aim of this study was to conduct a randomised controlled trial to evaluate the comparative effectiveness of transitional care interventions on reducing unplanned hospital readmissions and health service use, functional ability, psychosocial well-being and cost-effectiveness of care. This paper reports results on unplanned hospital readmissions within 28 days, at 12 weeks and at 24 weeks following hospital discharge.

## Methods

### Design

Randomised controlled trial to determine the comparative effectiveness of transitional care interventions on prevention of unplanned hospital readmissions in high-risk older adults.

### Participants

All patients admitted to any medical ward in two tertiary metropolitan hospitals were screened for eligibility. Inclusion criteria for the study were adults aged 65 years or over, admitted with a medical condition and who had at least one risk factor for readmission. Risk factors for readmission have been previously identified in the literature and were utilised for inclusion criteria [14–16], including age of 75 or older, more than one hospital admission in previous 6 months, multiple comorbidities, living alone, poor social support, poor self-rating of health, functional impairment and/or a history of depression. Exclusion criteria were requiring home oxygen, dependence on a wheelchair or unable to walk independently for 3 m (independently was defined as able to walk without other human aid, whether using a mechanical aid or not), living in a nursing home, or presence of a cognitive deficit or progressive neurological disease. The study aimed to evaluate transitional care interventions for older adults known to be at high risk of hospital readmission, yet still with potential to respond well to early intervention.

### Procedure

Ethical approval was obtained from the participating organisations’ Human Research Ethics Committees (Human Research Ethics Committees of the Mater Health Services {No. 1173A} and Queensland University of Technology {No. 0800000219}) and complied with the Declaration of Helsinki rules for human experimentation. Written consent was obtained from all participants. Recruitment and data collection occurred from 2008 to 2011.

Within 72 h of admission, eligible patients were invited to participate, written informed consent obtained, and baseline data collected. Following collection of baseline data, the research assistant opened a sealed sequential randomisation envelope, which had been prepared prior to commencement of recruitment by the Project Coordinator via a computerised randomised program. The participant was then randomised to one of the four groups: 1) usual care, 2) exercise program, 3) nurse home visit and telephone follow-up (N-HaT), or 4) exercise and nurse home visit and telephone follow-up (ExN-HaT). Participants in the control group received routine hospital and follow-up care as provided by the health service. This involved a needs assessment by the hospital health staff, discharge planning, and referrals for follow up services as appropriate.

In addition to usual care, participants in the ExN-HaT group received an assessment and tailored exercise program (taking approximately two hours) and six weekly in-home follow-up visits by an exercise physiologist, requiring around two hours per visit. This was combined with an in-home visit within 48 h of discharge (~two hours) and regular telephone follow-up (~ 30 min/call) for 24 weeks by a gerontic nurse (weekly for the first 4 weeks, then every 4 weeks, or more frequently as required). The exercise group, in addition to usual care, received the tailored exercise program and six-weekly in-home follow-up visits by an exercise physiologist; whilst the N-HaT follow-up group, in addition to usual care, received only the in-home visit within 48 h of discharge and regular telephone follow-up for 24 weeks by a gerontic nurse. Detailed information on the intervention protocol is published in Courtney et al. (2011) [17].

### Data collection and measures

The primary outcome measure was unplanned hospital readmission. Although there is variation in the definition of unplanned readmissions [18], for the purpose of this study and consistency with health system definitions, an unplanned readmission is defined as any admission (all cause) for an unforeseen or unplanned (non-elective) cause within 24 weeks of discharge from an index acute hospital admission. This was determined by audit of the hospital records by an independent person to the project.

Participants completed a questionnaire at baseline within 72 h of hospital admission on socio-demographics, functional ability (Instrumental Activities of Daily Living [19], Walking Impairment Questionnaire [20]) and psychosocial well-being (Geriatric Depression Scale [21], MOS Social Support Survey [22], SF-12 [23]). Full details of the instruments are available in Courtney et al. [17] Socio-demographic data included age, gender, education, employment status, income, living arrangements, and hospital insurance status. Data on diagnoses, health and medical history was obtained from medical records.

A telephone interview was conducted at 28 days, 12 weeks and 24 weeks following hospital discharge with all participants from all groups by an independent research assistant with post-graduate qualifications in health, who was blinded to group allocation, to gather data on follow up measures of psychosocial well-being and functional ability, and post-discharge health service. Information on all-cause unplanned hospitalisations was obtained from both the participants during interviews, and from hospital medical records. Hospital records data were abstracted by the medical records department at the hospital, by independent staff unknown to the study. Data on adherence to the intervention program and achievement of goals were assessed and recorded during each intervention follow-up, i.e. during the 6 weekly visits for participants involved in the exercise groups, and/or during telephone follow-up calls during the 24 weeks following discharge for the participants receiving the N-HaT intervention. Adherence to chronic disease management strategies or goals was assessed during the nurse telephone follow-up calls and progress recorded qualitatively. Adherence to the home-based exercises programme was defined as undertaking the recommended exercise intervention for greater than or equal to 75% of the time. Adherence to the exercise program was low with a range of 42–68% of participants adhering to the exercises over the 24 weeks. This paper reports intention to treat outcomes, and adherence to protocol outcomes will be reported separately.

### Statistical analysis

Descriptive statistics were calculated for all variables. The pattern of missing data was checking by testing differences between cases with missing data and cases with no missing data and no significant differences were found. All data analyses were conducted based on the principle of intention to treat [24]. Chi square, ANOVA, and Kruskal-Wallis tests were used for bivariate analysis of differences between groups. For the primary outcome of unplanned hospital readmissions, Chi square analysis and Kaplan-Meier survival curves were used to compare the three intervention groups and control group at the bivariate level, while Cox proportional hazards regression models were used to determine the independent effect of the interventions.

## Results

**Fig. 1:**
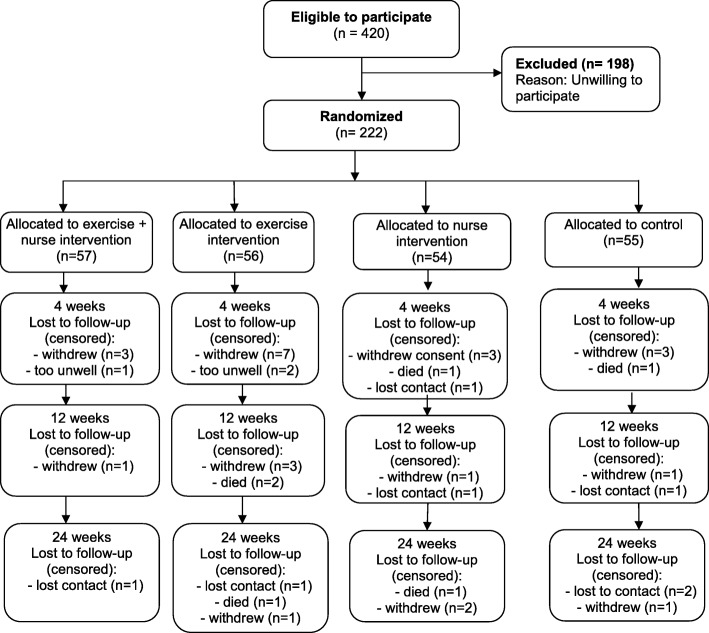
Flow of participants through study

A sample of 222 patients was recruited (55 in the control group, 56 in the exercise only intervention group, 54 in the N-HaT intervention group, and 57 in the ExN-HaT intervention group). The Consort flow diagram of participants through the study is shown in Fig. 1. Across the 24 week intervention period, 39 participants dropped out due to deterioration in health, death, changed address or withdrawn consent. All participants were under the supervision of their medical team and deteriorations were handled by their clinicians. A comparison of demographic characteristics, admission diagnosis, length of hospital stay, risk factors, and functional ability scores between those who left the study and those who continued revealed no significant differences between the study groups at baselines. However, participants who discontinued in the program had significantly higher rates of co-existing renal disease (Chi^2^ 5.94, *p* = 0.015) and less social support (*t* = 2.14, *p* = 0.037).

### Demographic and medical information

Baseline demographic characteristics, admission diagnoses, comorbidities, and risk factors for readmission according to group are displayed in Table 1. More women (73%, *n* = 162) than men (27%, *n* = 60) participated, with the average age being 77.6 years (SD = 6.64, range 65–93 years). Respiratory disease (38%, *n* = 83) and cardiac disease (23%, *n* = 50) were the most frequent diagnoses on admission. The median number of co-morbidities was four (range 1–9), the most common being cardiac disease (83%, *n* = 184), orthopaedic conditions (57%, *n* = 126), and respiratory disease (56%, *n* = 125). The median duration of hospital stay was 5 days (range 1–47 days). The majority of participants (94%, *n* = 207) had multiple risk factors for readmission with a median number of three (range 1–7), most frequently multiple co-morbidities (95%, *n* = 211), age over 75 years (66%, *n* = 146), and living alone (48%, *n* = 106). There were no significant differences between the groups with regard to demographic variables, diagnosis, co-morbidities, risk factors, or length of hospital stay. There was a total of nine planned routine hospital admissions during the study period – one in the control group, 2 in the exercise only group, 3 in the N-HaT group and 4 in the ExN-HaT group; for colonoscopies, gastroscopies, orthopaedic surgery, pacemaker insertion and one skin graft.

**Table 1:** Demographics, diagnoses, co-morbidities, risk factors for readmission

Characteristic	Group				Total
	EN-HaT	Exercise	N-HaT	Control	
Group, n	57	56	54	55	222
Demographic Details					
Age, M ± SD	77.1 (7.64)	77.6 (6.50)	77.8 (6.23)	77.9 (6.20)	77.6 (6.64)
Female, n(%)	46 (80.7)	42 (75.0)	37 (68.5)	37 (67.3)	162 (73.0)
Admission Diagnosis, n (%)					
Respiratory disease	28 (49.1)	21 (37.5)	17 (31.5)	17 (30.9)	83 (37.4)
Cardiac disease	12 (21.1)	13 (23.2)	13 (24.1)	12 (21.8)	50 (22.5)
Renal	4 (7.0)	3 (5.4)	3 (5.6)	3 (5.5)	13 (5.9)
Falls	1 (1.8)	3 (5.5)	4 (7.4)	1 (1.8)	9 (4.1)
Other	11 (19.3)	15 (26.8)	17 (31.5)	21 (38.1)	64 (28.8)
Comorbidities, n (%)					
Cardiovascular disease	49 (85.9)	54 (96.4)	54 (100)	47 (85.5)	204 (91.8)
Orthopaedic	35 (61.4)	28 (50.0)	32 (59.3)	31 (56.4)	126 (56.8)
Respiratory disease	33 (57.9)	35 (62.5)	29 (53.7)	28 (50.9)	125 (56.3)
Gastrointestinal	32 (56.1)	23 (41.1)	30 (55.6)	30 (55.6)	115 (51.8)
Endocrine	14 (24.6)	18 (32.1)	20 (37.0)	11 (20.0)	63 (28.4)
Renal	9 (15.8)	12 (21.4)	11 (20.4)	11 (20.0)	43 (19.4)
Other	29 (50.1)	27 (48.2)	22 (40.7)	30 (54.5)	108 (48.6)
Number of comorbidities	median (range)				
	4 (1–8)	4 (1–8)	4 (1–9)	4 (1–8)	4 (1–9)
Length of hospital stay	5 (1–13)	6.77 (6.27)	5.09 (3.81)	5 (1–34)	5 (1–47)
Number of risk factors	3 (1–7)	3 (1–7)	4 (1–7)	3 (1–6)	3 (1–7)
Risk factors n (%)					
Multiple comorbidities	55 (96.5)	55 (98.2)	51 (94.4)	50 (90.9)	211 (95.0)
Age ≥ 75	36 (63.2)	35 (62.5)	37 (68.5)	38 (69.1)	146 (65.8)
Poor/fair health self-rating	31 (54.4)	34 (60.7)	23 (42.6)	24 (43.6)	112 (50.5)
Lived alone	25 (43.9)	29 (51.8)	27 (50.0)	25 (45.5)	106 (47.7)
Functional impairment	14 (24.6)	17 (30.4)	18 (33.3)	14 (25.5)	63 (28.4)
Admissions in 6 months	11 (19.3)	8 (14.3)	19 (35.2)	11 (20.0)	49 (22.1)
Admission in last 30 days	10 (17.5)	12 (21.4)	13 (24.1)	5 (9.1)	40 (18.0)
Poor social support	6 (10.5)	11 (21.4)	12 (22.2)	11 (20.0)	41 (18.5)
History of depression	7 (12.3)	7 (12.5)	8 (14.8)	4 (7.3)	26 (11.7)

### Unplanned readmissions in the 28 days following discharge

In the 28 days following discharge, 25% (13 of 53) of the control group, 14% (7 of 49) of the exercise only intervention group, 10% (5 of 49) of the N-HaT intervention group, and 8% (4 of 53) of the ExN-HaT intervention group experienced an unplanned hospital readmission (Chi square 6.75, *p* = 0.010).

**Fig. 2:**
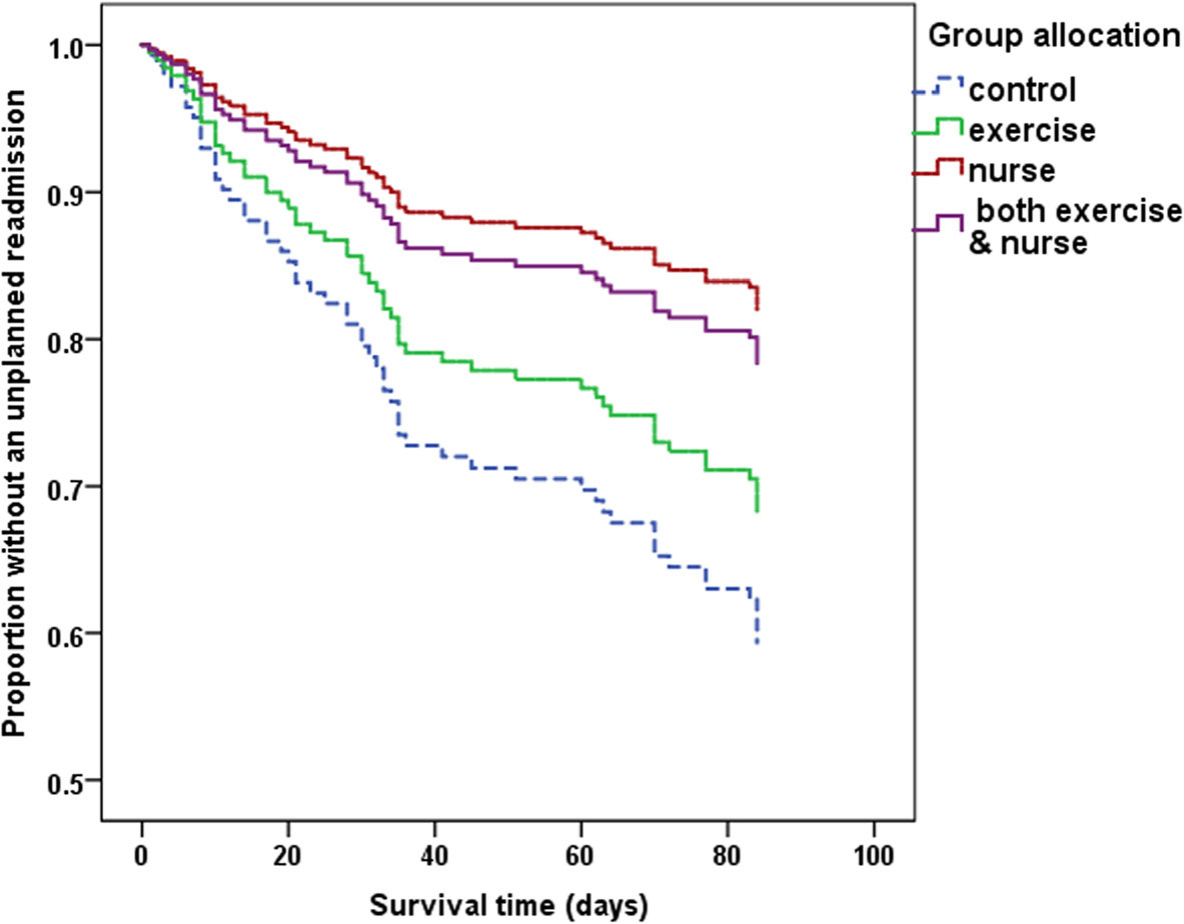
Unplanned readmissions within 84 days, Adjusted Survival Lines

All variables found to be associated with unplanned readmission at the bivariate level (*p* < 0.05, i.e. group, co-existing renal disease, depression, living alone, chronic disease management self-efficacy), or identified in the literature as having a significant effect on unplanned readmissions (age), were entered simultaneously into a Cox proportional hazards regression model using unplanned readmission in the 28 days as the dependent variable. After mutual adjustment for all variables, the model outcomes found that participants in the ExN-HaT intervention group were 3.6 times less likely to have an unplanned readmission than those in the control group (HR 0.278, 95% CI 0.09–0.87, *p* = 0.029, see Table 2). Participants in the N-HaT intervention group were 2.6 times less likely to be readmitted (HR 0.38, 95% CI 0.13–1.07, *p* = 0.067), and those in the exercise only intervention group were 1.99 times (HR 0.501, 95% CI 0.19–1.27, *p* = 0.148) less likely to be readmitted than the control group; however, neither of these two intervention groups were statistically significantly different from the control group (see Table 2). The adjusted survival curves are demonstrated in Fig. 2.

**Table 2:** Unplanned hospital readmissions in 28 days - Cox proportional hazards regression model

	β	Hazard Ratio	95% CI	p
Randomised Group				
Control group	referent group			
Exercise only group	−0.69	0.501	0.19–1.28	0.148
N-HaT^c^group	−0.97	0.379	0.13–1.07	0.067
ExN-HaT^d^group	−1.28	0.278	0.09–0.88	0.029
Co-existing renal disease	0.98	2.659	1.18–5.97	0.018
Geriatric Depression Scale^a^	0.18	1.191	1.03–1.37	0.017
CDM Self efficacy scale^b^	0.46	1.589	1.20–2.11	0.001

^a^Geriatric Depression Scale Short-Form, scale 0–15, where 0 = no depressive symptoms, and 15 = large number of depressive symptoms with a high risk of depression

^b^Chronic Disease Self Efficacy Scale [35] – Management of chronic disease sub-scale, where higher scores indicate higher levels of self efficacy

^c^Nurse Home/telephone follow-up

^d^ Exercise and Nurse Home/telephone follow-up

Participants with co-existing renal disease, higher Geriatric Depression Scale scores and higher scores on the Chronic Disease Management Self-Efficacy scale were also significantly more likely to have an unplanned readmission (see Table 2). The model was significant, Chi-square 21.7 (6), *p* < 0.001, − 2 Log Likelihood 274.4.

### Unplanned readmissions in the 12 weeks following discharge

For all-cause unplanned hospital readmissions in the longer term, by 12 weeks after discharge, 38% of the control group, 36% of the exercise only group, 19% of the N-HaT group, and 20% of the ExN-HaT group experienced an unplanned hospital readmission. The ExN-HaT and N-HaT groups had significantly lower unplanned readmissions than the control group (*p* = 0.049; *p* = 0.029 respectively, no difference between N-HaT and ExN-HaT groups).

All variables found to be associated with unplanned readmission at the bivariate level (*p* < 0.05, i.e. group, co-existing renal disease, living alone, functional impairment, total number of risk factors for readmission, WIQ Speed Subscale score), or identified in the literature as having a significant effect on unplanned readmissions (age), were entered simultaneously into a Cox proportional hazards regression model using time to unplanned readmission in the 12 weeks as the dependent variable. After mutual adjustment for all variables, participants in the ExN-HaT or the N-HaT intervention groups were 2.14 and 2.64 times, respectively less likely to suffer an unplanned hospital readmission in the 12 weeks after discharge (*p* = 0.040; *p* = 0.014 respectively), see Table 3. The model was significant, Chi-square 19.03 (7), *p* = 0.006, − 2 Log Likelihood 553.01.

**Table 3:** Unplanned hospital readmissions in 12 weeks after discharge - Cox proportional hazards regression model

	β	Hazard Ratio	95% CI	p
Randomised Group				
Control group	referent group			
Exercise only group	−0.30	0.74	0.37–1.47	0.385
N-HaT group^b^	− 0.97	0.38	0.18–0.82	0.014
ExN-HaT group^c^	− 0.76	0.47	0.23–0.97	0.040
Co-existing renal disease	0.41	1.51	0.81–2.83	0.198
Lives alone	0.53	1.70	0.90–3.21	0.099
Total number of risk factors	0.137	1.15	0.93–1.42	0.205
WIQ Speed Scale^a^	− 0.003	0.99	0.98–1.01	0.695

^a^Walking Impairment Questionnaire: Speed scale

^b^Nurse Home/telephone follow-up

^c^Exercise and Nurse Home/telephone follow-up

### Unplanned readmissions in the 24 weeks following discharge

By 24 weeks after discharge, there were no significant differences between groups, with 46% of the control group, 42% of the exercise only group, and 34% of the N-HaT and the ExN-HaT intervention groups experiencing an unplanned hospital readmission. All variables found to be associated with unplanned readmission at the bivariate level (*p* < 0.05, i.e. living alone, multiple comorbidities, WIQ Speed Subscale score, chronic disease management self-efficacy scale), or identified in the literature as having a significant effect on unplanned readmissions (age), were entered simultaneously into a Cox proportional hazards regression model using unplanned readmission in the 24 weeks as the dependent variable. After mutual adjustment for all variables, results found participants who lived alone were significantly more likely to have an unplanned readmission (HR 2.1, 95% CI 1.28–3.29, *p* = 0.003). In the intervention groups, participants in the ExN-HaT or the N-HaT intervention groups were 1.82 and 1.88 times, respectively less likely to suffer an unplanned hospital readmission; although this did not reach statistical significance (N-HaT group *p* = 0.053; ExN-HaT group *p* = 0.058).

## Discussion

This study found that readmission to hospital was significantly reduced for the combined exercise and nurse follow-up (ExN-HaT) intervention group within 28 days and 12 weeks after discharge. At 28 days, there was a 14% unplanned readmission rate in the ExN-HaT group in contrast to a 25% unplanned readmission rate in the control group. In comparison, the literature reports unplanned readmission rates of older adults in Australia and the Asia-Pacific area as between 25 and 46% [12, 25], similar to the findings from the control group in this study. Smaller non-significant differences by 24 weeks after discharge. It is postulated that the strength of effect did not last for 24 weeks because of less interaction with the intervention nurse in the last 12 weeks of the stud protocol. Systematic reviews have found no single intervention is effective in reducing unplanned hospital readmissions for older adults [9, 10]. Similarly, this study found that multi-component interventions were more effective than an exercise-only intervention, or routine care, in preventing unplanned hospital readmissions. The findings on the 28 day readmission rates in this study are broadly consistent with other studies on readmissions in that single interventions are not significantly effective [26, 27].

Other studies have evaluated single and combined interventions designed to reduce readmission rates for older patients in the first month after discharge [10, 28–30]. However, few studies have focused on the comparative effectiveness of each component of a multi-faceted intervention, in comparison to the combined components. This study’s results are consistent with a previous study by this group which evaluated a multi-component intervention for at-risk older people on unplanned health service utilization, finding the intervention group had significantly fewer unplanned readmissions than the control group receiving routine care; however, the comparative effectiveness of the individual components of the intervention was unknown [12].

In this study, the interventions aimed to provide a means of promoting health in order to reduce unplanned readmissions. Interventions to reduce hospital readmissions for older adults with chronic conditions are essential given that over a quarter of avoidable hospitalisations occur in this age group and two-thirds of avoidable hospital admissions are due to chronic conditions [29]. Continued research to support the findings of the current study that multifaceted interventions in transitional care are more efficient in reducing unplanned readmissions than single interventions seems warranted.

Results show that the exercise only intervention was ineffective in reducing readmissions without additional support. This finding may reflect the need for more regular contact with the exercise physiologist, resulting in less motivation and confidence in exercising safely. In contrast, the multifaceted interventions provide more engagement with patients, through the crucial role of a transitional-care nurse. The nurse was able to provide a continuous point of contact across hospital and home and provide information and assist in setting individual goals for health and chronic disease management. Importantly, they were also able to provide support and encouragement to engage in self-management and refer to appropriate support services if required. In comparison, the Aged Care Transition Program [28] evaluated the effectiveness of a national transitional care program for older adults with complex care needs and limited social support, involving care coordinators, home visits and telephone follow-up calls for up to 2 months after discharge. Participants (*n* = 4132) of this program had significantly fewer unplanned re-hospitalisations and emergency visits at 30 days and 180 days after discharge, with the effect decreasing over time [28], similarly to this study. In direct contrast, the Aged Care Transition Program [28] not only educated or assisted participants by telephone but supplemented telephone calls with in-person meetings for 1 to 2 months after discharge. Our study supports the need for programs such as these, or for expanded hospital outreach services providing at least 6 months follow-up care via multiple strategies, including home visits and telehealth.

Unplanned readmissions continued steadily over the 6 months after discharge in this study and were influenced by a number of factors. For example, co-existing renal disease and higher Geriatric Depression Scale and Chronic Disease Self-Efficacy scores were significant factors associated with increased readmissions at 28 days, whereas living alone was significantly associated with increased readmission rate at 24 weeks. Impaired renal function is a well-known risk factor for readmission to hospital [31, 32], whereas a systematic review identified a lack of recognition of depression in ED in older adults [33], however it is yet to be identified whether this is a consistent independent risk factor for readmission. A recent study [34] evaluating an intervention designed to empower patients in self-management demonstrated that improved self-efficacy for chronic disease management subsequently reduced hospital readmission and increased quality of life. However, this study surprisingly found the reverse – in that those with higher chronic disease management self-efficacy scores were more likely to have unplanned readmissions; although the scale used in this study differed from the measure in the study above [34]. Further research is required to shed light on this result. Similar to our findings, living alone has been identified in a previous prospective cohort study of 328 low-income older adults as an independent risk factor for early readmission [16]. Future interventions should consider this risk factor as it can be identified easily at hospital admission; therefore, individuals at risk for early readmission may be targeted for interventions delivered during the hospital stay.

### Limitations

There are several limitations to the study. Neither the participants nor the intervention nurse or exercise physiologist were blinded to randomisation. However, the research assistant collecting the outcome data via telephone interviewing at 28 days, and 12 and 24 weeks was independent and blinded to groups. In addition, hospital data were retrieved from medical records and baseline data was collected before randomisation. Second, the desired sample size was not achieved within the study timeline. This may have resulted in a lack of power to detect a significant impact on readmissions at the 24 week time-point. Third, almost half of the eligible sample were unwilling to participate (Fig. 1). Older adults with risk factors for readmission, by default, are a frail population, and were cautious about volunteering for a potential exercise intervention. As yet, there has been no cost-effectiveness analysis of the interventions from this study’s data, however, the study was based on an earlier two-group study, with a control and combined intervention group (i.e. equivalent to ExNH-HaT group), which found the combined intervention was cost-effective.^12^ There is a need for further evaluation of the individual interventions.

## Conclusion

This study suggests that multifaceted transitional interventions for older adults at risk of hospital readmission can significantly reduce hospital readmissions within 28 days and 12 weeks of discharge. Greater understanding of the factors that influence unplanned readmission will contribute to the further development of interventions for transitional care.

## Abbreviations

ExN-HaT: Exercise program and Nurse Home visit and Telephone follow-up
N-HaT: Nurse Home visit and Telephone follow-up
SF-12: 12-item Short Form Survey
